# Cryptotanshinone Alleviates Cardiac Hypertrophy Through SIRT3-Mediated Epigenetic Regulation and Mitochondrial Dynamics

**DOI:** 10.3390/antiox15101329

**Published:** 2026-10-15

**Authors:** Jianming Xing, Peng Ji, Xintong Gu, Shuoxi Guo, Meilun Wang, Xushuang Jia, Jiaqi Chen, Junjie Chang, Xiaohan Cui, Ye Jin, Haicheng Gao, Da Liu

**Affiliations:** 1 Department of Clinical Pharmacy, School of Pharmaceutical Sciences, Jilin University, Changchun 130021, China; xingjm22@mails.jlu.edu.cn; 2 Public Laboratory Center, Changchun University of Chinese Medicine, Changchun 130117, China; 3 Department of Pharmacy, Changchun University of Chinese Medicine, Changchun 130117, China; 4 Northeast Asian Institute of Traditional Chinese Medicine, Changchun University of Chinese Medicine, Changchun 130117, China

**Keywords:** cryptotanshinone, *Salvia miltiorrhiza Bunge*, cardiac hypertrophy, mitochondrial dynamics, epigenetics

## Abstract

Cryptotanshinone (CTS), a bioactive diterpenoid extracted from *Salvia miltiorrhiza Bunge*, has long been recognized as a traditional Chinese herbal compound. This study reveals that CTS exerts potent anti-hypertrophic effects against angiotensin II (Ang II)-induced cardiac hypertrophy by targeting Sirtuin 3 (SIRT3)-mediated epigenetic and mitochondrial pathways. In both in vitro and in vivo models, CTS ameliorated pathological hypertrophy, oxidative stress, inflammation, and apoptosis. Mechanistically, CTS upregulated SIRT3, reducing histone H3K9 acetylation (H3K9ac) and restoring mitochondrial dynamics by balancing fission (DRP1) and fusion (MFN2) proteins. Genetic ablation of SIRT3 exacerbated hypertrophy and mitochondrial dysfunction, while pharmacological inhibition with 3-TYP abolished CTS benefits. These findings elucidate CTS as a therapeutic candidate for cardiac hypertrophy by activating SIRT3, which is accompanied by reduced H3K9ac and restored DRP1/MFN2 balance, highlighting potential in cardiac hypertrophy treatment.

## 1. Introduction

Cardiac hypertrophy represents a critical transitional pathological state that frequently progresses to heart failure and sudden cardiac death, constituting a severe threat to global public health [1]. Mechanistically, hypertrophic stimuli trigger intricate intracellular signaling pathways that reactivate fetal gene expression programs, initiating cardiac hypertrophy and left ventricular remodeling processes that invariably advance toward terminal heart failure [2]. Among these pathways, angiotensin II (Ang II), as a core component of the renin–angiotensin–aldosterone system, mediates cellular phenotypic transformation and morphological changes. It stimulates excessive reactive oxygen species (ROS) production, induces inflammatory responses and platelet activation, ultimately contributing to hypertension, atherosclerosis and heart failure pathogenesis [3]. Notably, cardiac dysfunction consistently involves mitochondrial metabolic disorders, making modulation of mitochondrial metabolism-related proteins and enzymes a promising therapeutic strategy for cardiac hypertrophy [4,5].

Danshen (*Salvia miltiorrhiza Bunge*), derived from the dried roots and rhizomes of *Salvia miltiorrhiza*, holds cardioprotection, anti-inflammation, and antioxidation properties [6]. Furthermore, cryptotanshinone (CTS), as the principal liposoluble extract of Danshen, modulates multiple signaling pathways including JAK2/STAT3, PI3K/AKT, and AMPK, thereby inhibiting cellular proliferation, migration and invasion while promoting autophagy and apoptosis [7]. Although CTS significantly alleviates myocardial ischemia–reperfusion injury, its potential impact on myocardial hypertrophy remains unexplored. Mitochondria occupy a central position in cardiac remodeling processes, serving not only as the primary sites of cellular energy metabolism but also as crucial regulators of calcium homeostasis, apoptosis and gene expression [8]. Mitochondrial dynamics involve precisely balanced fusion and fission processes: mitofusin 2 (MFN2) mediates mitochondrial fusion, while dynamin-related protein 1 (DRP1) regulates mitochondrial fission [9]. Disruptions in mitochondrial dynamics impair energy metabolism and calcium handling, directly contributing to cardiac hypertrophy and heart failure progression [10]. Emerging evidence indicates that promoting mitochondrial fusion while suppressing excessive fission reduces ROS accumulation and improves contractile dysfunction, thereby preventing the transition from compensatory hypertrophy to decompensated heart failure [11,12]. Sirtuin 3 (SIRT3), a class III mitochondrial NAD^+^-dependent protein deacetylase, regulates mitochondrial protein function through lysine deacetylation modifications, thereby maintaining redox balance, electron transport chain activity and fatty acid oxidation [13]. What is more, SIRT3 mediated the enhancement of complex I activity promotes ATP production and improves cardiomyocyte contractile function [14]. Beyond mitochondrial regulation, SIRT3 also modulates gene expression through deacetylation of histone H3 at lysine 9 (H3K9), influencing cellular functional remodeling during cardiac hypertrophy development [15,16].

Based on these established mechanisms, we hypothesize that CTS may attenuate Ang II-induced cardiomyocyte hypertrophy by activating SIRT3-mediated H3K9 acetylation (H3K9ac) regulation, restoring the DRP1/MFN2 balance and reestablishing mitochondrial dynamic homeostasis, potentially involving coordination with other signaling pathways. The current study systematically investigates the mechanistic role of the CTS in cardiac hypertrophy, aiming to provide novel theoretical foundations and therapeutic targets for preventing and treating heart failure.

## 2. Materials and Methods

### 2.1. Reagents and Materials

H9C2 cardiomyocytes were obtained from Procell Life Science & Technology (Wuhan, China). Dulbecco’s modified Eagle’s medium (DMEM) was acquired from Thermo Fisher Scientific (Waltham, MA, USA). Phosphate-buffered saline (PBS), fetal bovine serum (FBS), penicillin–streptomycin solution, and the cell counting kit-8 (CCK-8) were provided by Solarbio Science & Technology (Beijing, China). Cryptotanshinone (Cat. No. IC0590; CAS No. 35825-57-1; Molecular formula: C_19_H_20_O_3_ Purity: HPLC ≥ 98%) and angiotensin II (Cat. No. SA5820; CAS No. 4474-91-3) were supplied by Beijing Solarbio Science & Technology. DAPI dye (Cat. No. AS21L123) was purchased from Life-iLab Biotech Co., Ltd. (Shanghai, China). Enzyme-linked immunosorbent assay (ELISA) kits for interleukin-6 (IL-6), interleukin-1β (IL-1β), tumor necrosis factor-α (TNF-α), malondialdehyde (MDA), glutathione peroxidase (GSH-Px), atrial natriuretic peptide (ANP), brain natriuretic peptide (BNP), and ATP were purchased from Jiangsu Meimian Industrial Co., Ltd. (Yancheng, China). ELISA kits for Mitofusin 2 (MFN2) and Dynamin-related protein 1 (DRP1) were purchased from Quanzhou Ruixin Biological Technology Co., Ltd. (Quanzhou, China). Phalloidin-iFluor 488 Reagent (ab176753) was obtained from Abcam (Waltham, MA, USA). Antibodies against ANP (Cat. No. 27426-1-AP), BNP (Cat. No. 28214-1-AP), SIRT3 (Cat. No. 10099-1-AP), H3K9ac (Cat. No. 39137), DRP1 (Cat. No. 12957-1-AP), MFN2 (Cat. No. 12186-1-AP), Bax (Cat. No. 50599-2-Ig), Bcl-2 (Cat. 68103-1-Ig), caspase-3 (Cat. No. 19677-1-AP), GAPDH (Cat. No. 60004-1-Ig), and β-actin (Cat. No. 66009-1-Ig) were obtained from Proteintech (Wuhan, China). 3-TYP (Cat. No. S8628) was supplied by Selleck Chemicals (Houston, TX, USA).

### 2.2. Cell Culture and Treatment

H9C2 cells were cultured in DMEM high-glucose medium supplemented with 10% FBS and 1% Penicillin–Streptomycin under an atmosphere of 5% CO_2_ at 37 °C. For cardiac hypertrophy model establishment, cells were stimulated with 1 µmol/L Ang II [17]. In addition, 5 μM of 3-TYP was administered to inhibit SIRT3 [18]. CTS (5, 10, 15 μM) was added to the medium for treatment. After 24 h, cells were collected for further analysis.

### 2.3. Establishment of the Mouse Cardiac Hypertrophy Model

Eight-week-old male C57BL/6J mice (20–25 g) were procured from Liaoning Changsheng Biotechnology (Shenyang, China). Cardiac hypertrophy was induced by subcutaneous injection of angiotensin II (1.5 mg/kg) with an osmotic pump for 14 days [19]. After intervention, successful model establishment was confirmed by echocardiographic analyses. Mice were anesthetized with 3% isoflurane inhalation and examined by two-dimensional guided M-mode echocardiography using a Technos MPX ultrasound system (Esaote, Genova, Italy) equipped with a Vevo 2100 imaging platform. The successfully established mouse model was randomly divided into four groups, each containing eight mice. The drug was administered via intraperitoneal injection for 14 days. Control group: PBS; CTS group: 25 mg/kg, 50 mg/kg, and 70 mg/kg CTS. At termination, the animals were euthanized using carbon dioxide asphyxiation, and serum and major organs were collected. After the cardiac tissues were fixed and embedded in paraffin, they were subjected to H&E and Masson’s stain [20], and ImageJ 1.53k software (NIH, Bethesda, MD, USA) was used to perform quantitative collagen analysis of Masson’s stain results.

### 2.4. Quantitative Real-Time PCR

Total RNA was isolated using TRIzol reagent. First-strand cDNA synthesis was performed using a PrimeScript RT reagent kit (Takara, Shiga, Japan). Quantitative PCR was carried out on a Real-Time PCR Detection System (Bio-Rad CFX96, Hercules, CA, USA) with SYBR Green Master Mix (Takara, Shiga, Japan). TCycle conditions: denature at 95 °C for 30 s, followed by 40 cycles, each consisting of 5 s at 95 °C and 30 s at 60 °C. Relative gene expression was normalized to GAPDH and calculated by the 2^–ΔΔCt^. The primer sequences are shown in Supplementary Table S1.

### 2.5. Construction of the pX459-sgRNA Plasmid for SIRT3 Gene Knockout

Construction of SIRT3-knockout plasmids using Single-guide RNA (sgRNA) sequences targeting mouse SIRT3 were designed using the CRISPR design tool provided by the Broad Institute and validated against the NCBI reference sequence. Two sgRNA pairs were synthesized (Supplementary Table S2). The pX459 vector was linearized with BbsI (NEB) at 37 °C for 30 min. Annealed sgRNA duplexes were ligated into the linearized pX459 backbone using T4 DNA ligase (Takara, Shiga, Japan) at 16 °C for 1 h. Competent ‘*Escherichia coli*’ DH5α cells were transfected with the products, and the positive clones were selected on ampicillin-containing LB agar. Plasmids were confirmed by Sanger sequencing (GENEWIZ, Suzhou, China).

### 2.6. Actin Staining with Phalloidin

The treated H9C2 cells were first fixed with 4% paraformaldehyde for 15 min. Subsequently, the cell membranes were permeabilized with 0.1% Triton X-100 for 10 min, and finally blocked with 1% bovine serum albumin for 30 min. Actin filaments were labeled with Phalloidin-iFluor 488 Reagent (1:200) for 45 min, while the cell nuclei were stained with DAPI (1 µg/mL). Images were captured, and three different fields of view were selected at random. The area of actin was measured using ImageJ 1.53k software (NIH, Bethesda, MD, USA) and average area per cell was then calculated.

### 2.7. Apoptosis Analysis

Apoptosis was assessed using an Annexin V-FITC/PI detection kit (E-CK-A211, Elabscience, Wuhan, China). After treatment, H9C2 cells were washed with PBS and then incubated with 5 µL of Annexin V-FITC and 5 µL of PI for 15 min. Apoptosis was evaluated immediately using a Beckman Coulter CytoFLEX flow cytometer (CA, USA), and the results were analyzed by CytExpert 2.4.0.28software (Beckman, Brea, CA, USA).

### 2.8. Mitochondrial Membrane Potential Assay

Mitochondrial membrane potential (ΔΨm) was evaluated using the JC-1 probe (AC12L073, Life-iLab Biotech, Shanghai, China). Cells were incubated with JC-1 staining solution (37 °C, 10 min) and washed with JC-1 buffer. Finally, cells were tested by flow cytometry (CytoFLEX A00-1-1102, Beckman, Brea, CA, USA), and the results were analyzed by CytExpert 2.4.0.28 software (Beckman, Brea, CA, USA).

### 2.9. Reactive Oxygen Species (ROS) Measurement

Reactive oxygen species (ROS) levels in H9C2 cells were detected using the DCFH-DA probe (E-BC-K138-F, Elabscience, Wuhan, China). Specifically, after drug treatment, 10 µM DCFH-DA was added to the cells, which were then incubated at 37 °C for 20 min. The cells were washed three times with PBS and analyzed by flow cytometry (CytoFLEX A00-1-1102, Beckman, Brea, CA, USA), and the results were analyzed by CytExpert 2.4.0.28 software (Beckman, Brea, CA, USA).

### 2.10. Transmission Electron Microscopy (TEM)

Cells were first fixed in 2.5% glutaraltydehyde at 4 °C for 1.5 h, followed by post-fixation with 1% osmium tetroxide for 1 h. Cells were then dehydrated in ethanol, and embedded in Epon 812 resin to prepare 70 nm ultrathin sections. The sections were stained with 2% uranyl acetate and lead citrate, and finally imaged using a transmission electron microscope (JEM-1400 Plus, Tokyo, Japan).

### 2.11. Western Blot

Total proteins from H9C2 cells were extracted using RIPA lysis buffer (with protease/phosphatase inhibitors) and quantified via BCA assay. After separation by SDS-PAGE, the proteins were transferred to a PVDF membrane (Merck, Rahway, NJ, USA). The membranes were then blocked with 5% non-fat milk for 1 h. For first antibody conjunction, membranes were incubated with primary antibodies: β-actin (1:5000), ANP (1:5000), BNP (1:5000), H3K9ac (1:800), SIRT3 (1:5000), Bax (1:2000), Bcl-2 (1:2000), caspase-3 (1:3000), DRP1 (1:5000), and MFN2 (1:5000) overnight at 4 °C. Finally, membranes were incubated with secondary antibodies (HRP-conjugated anti-mouse/rabbit, 1:5000) for 1 h at room temperature. Protein bands were imaged and quantified using ImageJ 1.53k software (NIH, Bethesda, MD, USA).

### 2.12. Molecular Docking

The structure of CTS (CID: 160254) was obtained from the PubChem database (https://pubchem.ncbi.nlm.nih.gov/), while the structure of SIRT3 protein (ID: pdb_00003gls) was from the PDB database (http://www.rcsb.org/). Water and ligand were removed with PyMOL 3.1 software (Schrödinger Inc., New York, NY, USA), then converted to pdbqt format using Auto Dock 4.2.6 software (Scripps Research Institute, La Jolla, CA, USA). Finally, the results were visualized and binding energies were calculated using PyMOL 3.1 sofyware (Schrödinger Inc., New York, NY, USA).

### 2.13. Statistical Analysis

Data are expressed as mean ± SD. Statistical significance was determined by one-way ANOVA with Tukey’s multiple comparison test or unpaired two-tailed Student’s *t*-test. A *p*-value < 0.05 was considered statistically significant.

## 3. Results

### 3.1. Cryptotanshinone Attenuates Angiotensin II-Induced Cardiac Hypertrophy In Vivo

Natural bioactive compounds have emerged as an important source of cardiovascular therapeutics due to their pleiotropic pharmacological effects and superior safety profiles [21]. Among these, cryptotanshinone (CTS)—a bioactive compound isolated from *Salvia miltiorrhiza Bunge*—has garnered increasing interest for its cardioprotective potential [21]. Previous reports have demonstrated that CTS exerts anti-inflammatory and anti-apoptotic activities, and shows protective effects in atherosclerosis and dyslipidemias models [22,23]. However, its role in regulating cardiac hypertrophy remains unclear. Therefore, we conducted a systematic evaluation of this issue.

**Figure 1:**
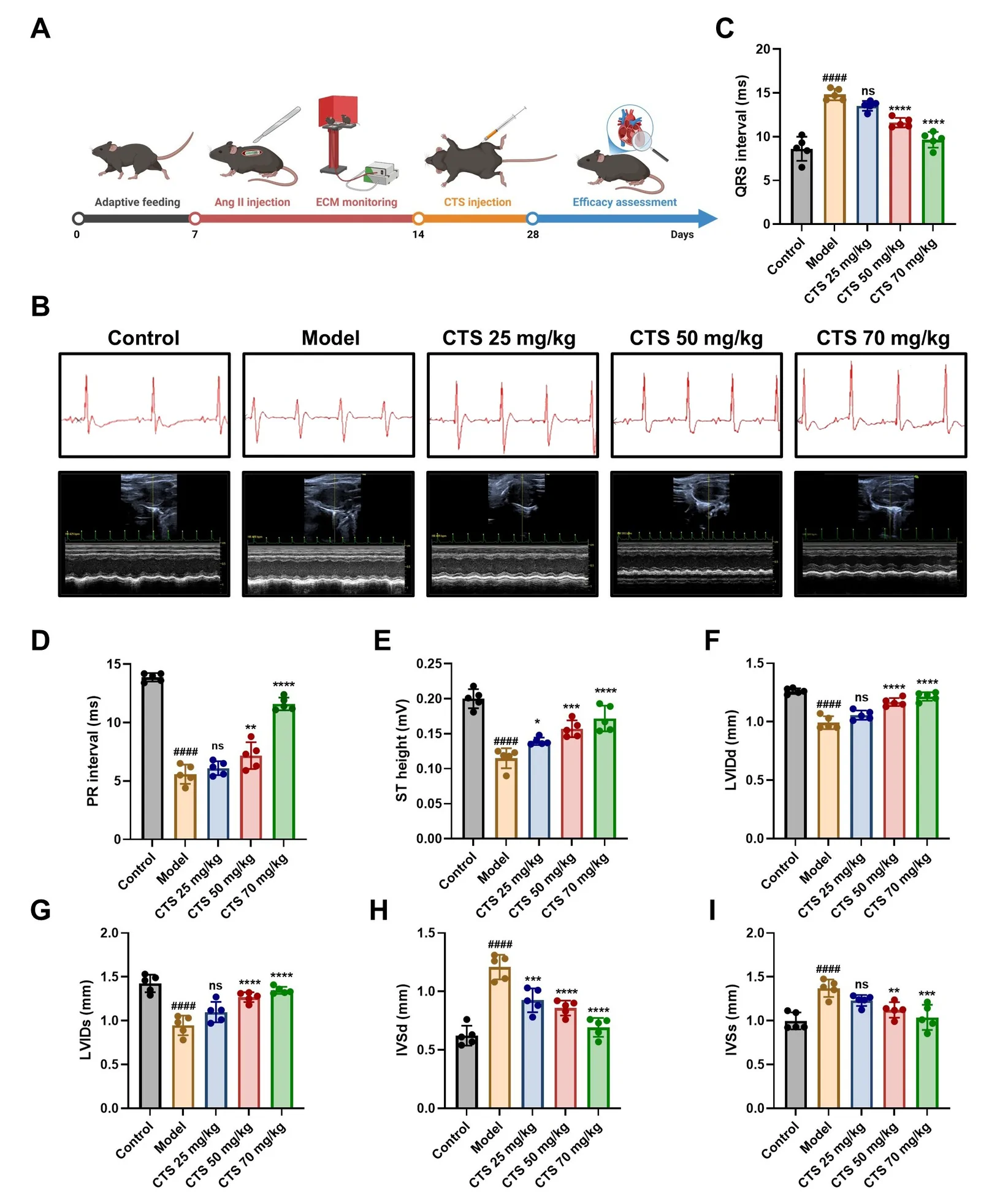
Cryptotanshinone alleviates physiological abnormalities caused by cardiac hypertrophy in vivo. (**A**) Schematic illustration of the cardiac hypertrophy model established by Ang II. (**B**) Electrocardiograms and echocardiograms of cardiac tissue in each group. QRS duration (**C**), PR interval (**D**), and ST height (**E**) of each group (*n* = 5). Echocardiographic assessment of each group (*n* = 5): LVIDd (**F**), LVIDs (**G**), IVSd (**H**), IVSs (**I**). Data are presented as mean ± standard deviation. #—significant difference versus control group; *—significant difference versus model group. * *p* < 0.05, ** *p* < 0.01, *** *p* < 0.001, ####/**** *p* < 0.0001, ns indicates no statistical significance. Created in BioRender. Xing, J. (2026) https://BioRender.com/hcqi0x2.

The Ang II-induced cardiac hypertrophy model was established to evaluate the therapeutic effects of CTS over a 14-day treatment period (Figure 1A). CTS administration produced significant improvements in the following electrical conduction parameters. Comprehensive cardiac phenotyping revealed that Ang II infusion induced substantial electrophysiological abnormalities (Figure 1B), where QRS duration was prolonged by 6.2 ms (Figure 1C), PR intervals reduced by 8.3 ms (Figure 1D), and ST height depression was lessened to 0.17 mV (Figure 1E)—hallmark features of pathological remodeling. CTS treatment markedly attenuated these alterations, reducing QRS prolongation by 5.18 ms, increasing PR intervals by 6.02 ms, and restoring ST height by 0.17 mV. Echocardiographic assessment demonstrated that Ang II induced characteristic structural changes, including decreased left ventricular internal dimensions during both diastole (LVIDd, Figure 1F) and systole (LVIDs, Figure 1G). What is more, Ang II stimulation significantly increased interventricular septal thickness in diastole (IVSd, Figure 1H) and systole (IVSs, Figure 1I). CTS treatment significantly reversed these structural changes: LVIDd and LVIDs were increased by 1.22 mm and 1.35 mm, respectively, while IVSd and IVSs were decreased by 0.69 mm and 1.04 mm, respectively. Consistent with functional improvements, quantitative analysis revealed that CTS dose-dependently reduced the heart-to-body weight ratio (HW/BW), with a maximal reduction of 27.63% at the highest dose compared with the Ang II group (Supplementary Figure S1).

**Figure 2:**
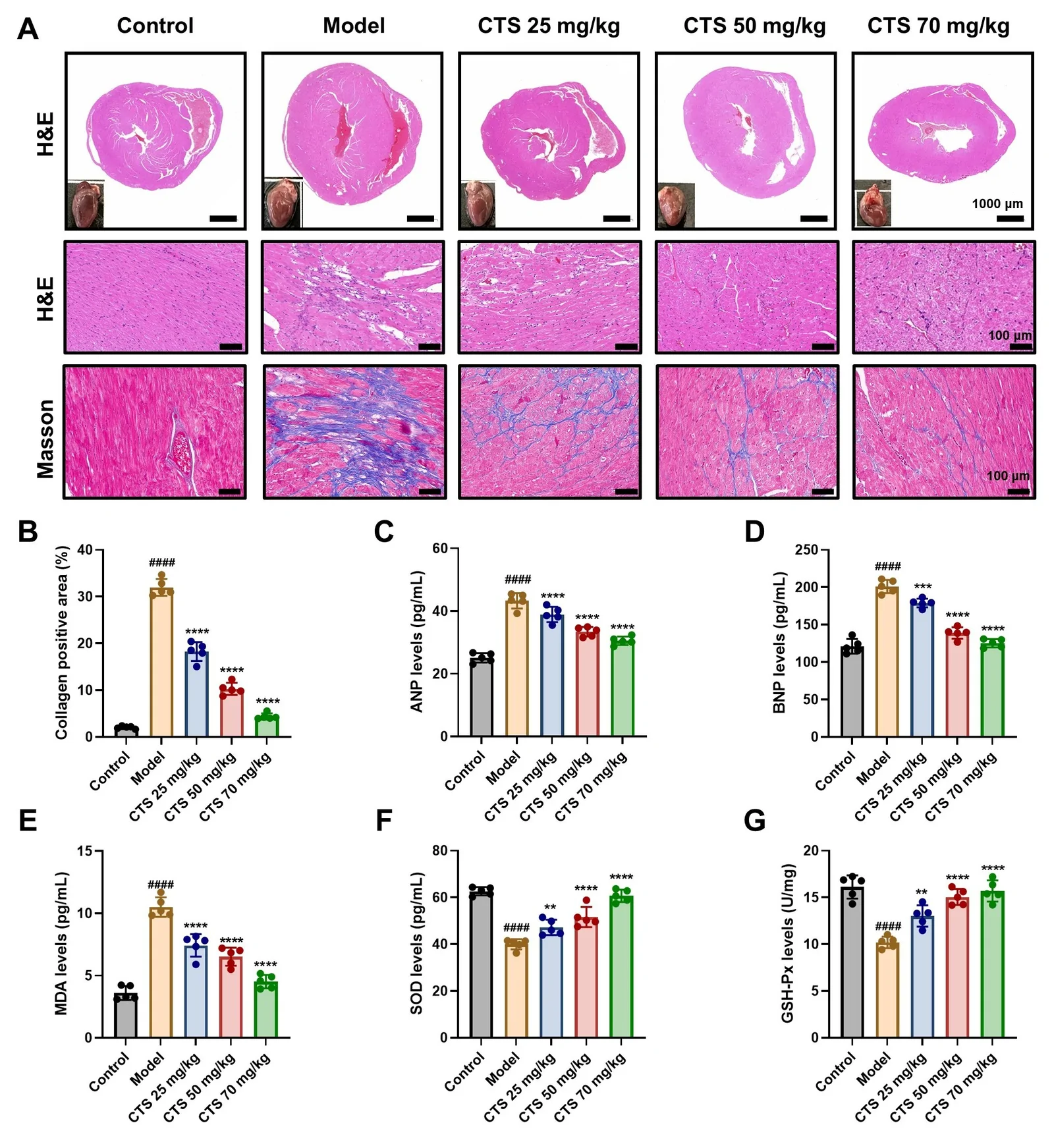
Cryptotanshinone alleviates abnormalities in tissue morphology and biological markers caused by cardiac hypertrophy in vivo. (**A**) H&E and Masson’s staining of cardiac tissue in each group. (**B**) Quantitative analysis of collagen volume (*n* = 5). ANP (**C**), BNP (**D**), MDA (**E**), SOD (**F**), and GSH-Px (**G**) levels in cardiac tissue homogenate (*n* = 5). Data are presented as mean ± standard deviation. #—significant difference versus control group; *—significant difference versus model group. ** *p* < 0.01, *** *p* < 0.001, ####/**** *p* < 0.0001.

Histopathological evaluation further demonstrated that CTS treatment ameliorated cardiac hypertrophy. H&E staining of liver, spleen, lung, and kidney showed no apparent morphological abnormalities, supporting the biosafety of CTS (Supplementary Figure S2). The heart images showed that CTS improved cardiomyocyte morphology, and restored myofibrillar organization, indicating preservation of myocardial architecture (Figure 2A). Masson’s staining of cardiac tissue confirmed that CTS inhibited fibroblast proliferation in a dose-dependent manner, reducing the fibrotic area from 31.97% in the Ang II group to 4.38% in the CTS-treated group (Figure 2A,B). Moreover, CTS significantly attenuated hypertrophy biomarker upregulation: atrial natriuretic peptide (ANP) expression was lowered by 29.47% (Figure 2C) and brain natriuretic peptide (BNP) by 37.74% (Figure 2D) compared with model levels. Mechanistic studies revealed that CTS administration restored redox homeostasis by reducing oxidative stress marker malondialdehyde (MDA, Figure 2E) while enhancing antioxidant defense superoxide dismutase (SOD, Figure 2F) and glutathione peroxidase (GSH-Px) activity (Figure 2G). CTS also exhibited potent anti-inflammatory effects, significantly suppressing the levels of pro-inflammatory cytokines TNF-α, IL-6, and IL-1β (Supplementary Figure S3). These findings collectively demonstrate that CTS effectively attenuates Ang II-induced cardiac hypertrophy through multimodal mechanisms involving electrical remodeling, structural preservation, oxidative stress mitigation, and inflammation suppression. CTS demonstrated significant therapeutic efficacy, supporting its potential as a novel therapeutic candidate for cardiac hypertrophy.

### 3.2. Cryptotanshinone Alleviates Cardiac Hypertrophy by Inhibiting Apoptosis

**Figure 3:**
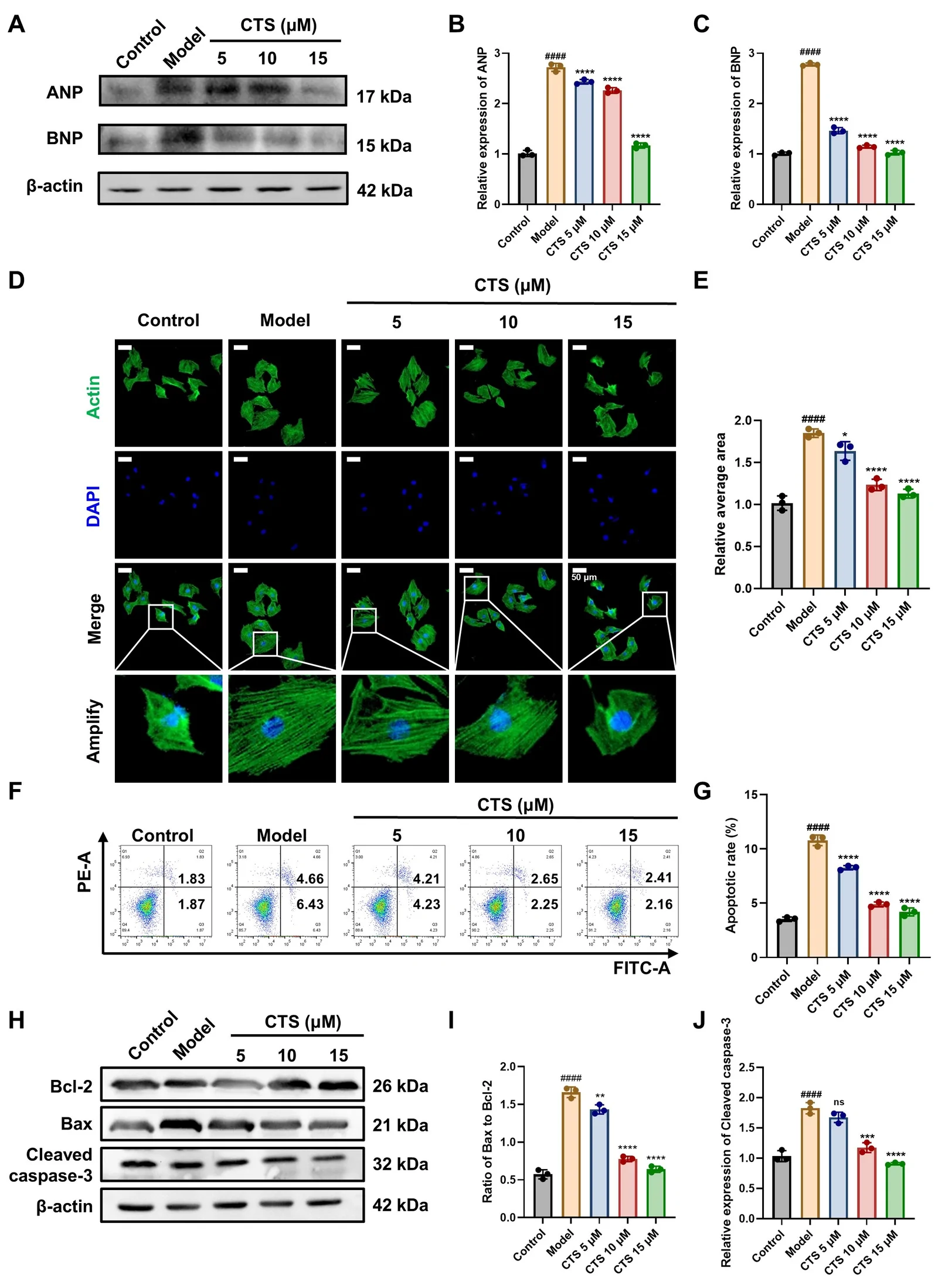
Cryptotanshinone alleviates cardiac hypertrophy by inhibiting apoptosis. (**A**) ANP and BNP expression in H9C2 cells measured by Western blot assay. Quantitative analysis of ANP (**B**) and BNP (**C**) levels using ImageJ software (*n* = 3). (**D**) H9C2 cardiomyocyte surface detected by Phalloidin-iFluor 488 Actin-Tracker fluorescent probes (green: actin, blue: nucleus). (**E**) Quantitative analysis of H9C2 cell surface area (*n* = 3). (**F**) Apoptosis of H9C2 cells analyzed by flow cytometry. (**G**) Apoptotic rate of H9C2 cells after treatment (*n* = 3). (**H**) Expression of Bcl-2, Bax, and Cleaved caspase-3 of H9C2 cells analyzed by Western blot assay. (**I**) Bax to Bcl-2 expression ratio (*n* = 3). (**J**) Quantitative analysis of Cleaved caspase-3 levels (*n* = 3). Data are presented as mean ± standard deviation. #—significant difference versus control group; *—significant difference versus model group. * *p* < 0.05, ** *p* < 0.01, *** *p* < 0.001, ####/**** *p* < 0.0001, ns indicates no statistical significance.

To evaluate the effects of CTS, a hypertrophy model was established in vitro by stimulating H9C2 cells with Ang II, after which graded doses of CTS were administered. Western blot analyses revealed that Ang II markedly upregulated ANP and BNP protein expression compared with control levels (Figure 3A). CTS dose-dependently suppressed this overexpression, reducing ANP by 57.11% (Figure 3B) and BNP by 63.03% (Figure 3C) relative to the Ang II group. The ELISA test confirmed this trend, showing parallel quantitative changes in secreted peptides (Supplementary Figure S4). Actin-Tracker probes further demonstrated that Ang II significantly increased cardiomyocyte surface area by 81.97% relative to controls, an effect that was reversed by CTS in a concentration-dependent manner, achieving a maximal surface area reduction of 38.92% (Figure 3D,E). Collectively, these data indicate that CTS effectively attenuates Ang II-induced cardiomyocyte hypertrophy, with effect sizes that scale with dosing. Ang II provokes cardiomyocyte injury by activating multiple apoptotic pathways, including mitochondrial dysfunction, culminating in hypertrophy, adverse remodeling and impaired cardiac function [24,25]. However, the mechanisms by which CTS modulates Ang II-induced apoptosis remain incompletely understood. We therefore conducted a series of experiments to clarify this issue. Hoechst staining revealed that Ang II increased nuclear condensation and apoptotic body formation—hallmarks of programmed cell death. CTS markedly inhibited this phenomenon (Supplementary Figure S5). Annexin V-FITC/PI probes confirmed that Ang II significantly elevated the total apoptosis rate, an effect that was dose-dependently attenuated by CTS, with the high-dose group showing a significant reduction (Figure 3F,G). Western blot assay demonstrated that Ang II upregulated the pro-apoptotic proteins Bax and cleaved caspase-3, while downregulating the anti-apoptotic protein Bcl-2 (Figure 3H). CTS treatment restored the balance among these proteins: at the highest dose, the ratio of Bax to Bcl-2 was lowered by 61.45% (Figure 3I), and cleaved caspase-3 was reduced by 50.24% (Figure 3J). In summary, CTS exerts a potent, dose-dependent anti-apoptotic effect against Ang II-induced cardiomyocyte injury, with quantitative improvements observed across multiple apoptotic indices. These findings support its potential as a cardioprotective agent and warrant further mechanistic and translational investigation.

### 3.3. Cryptotanshinone Suppresses Inflammation and Maintains Mitochondrial Membrane Potential Stability

**Figure 4:**
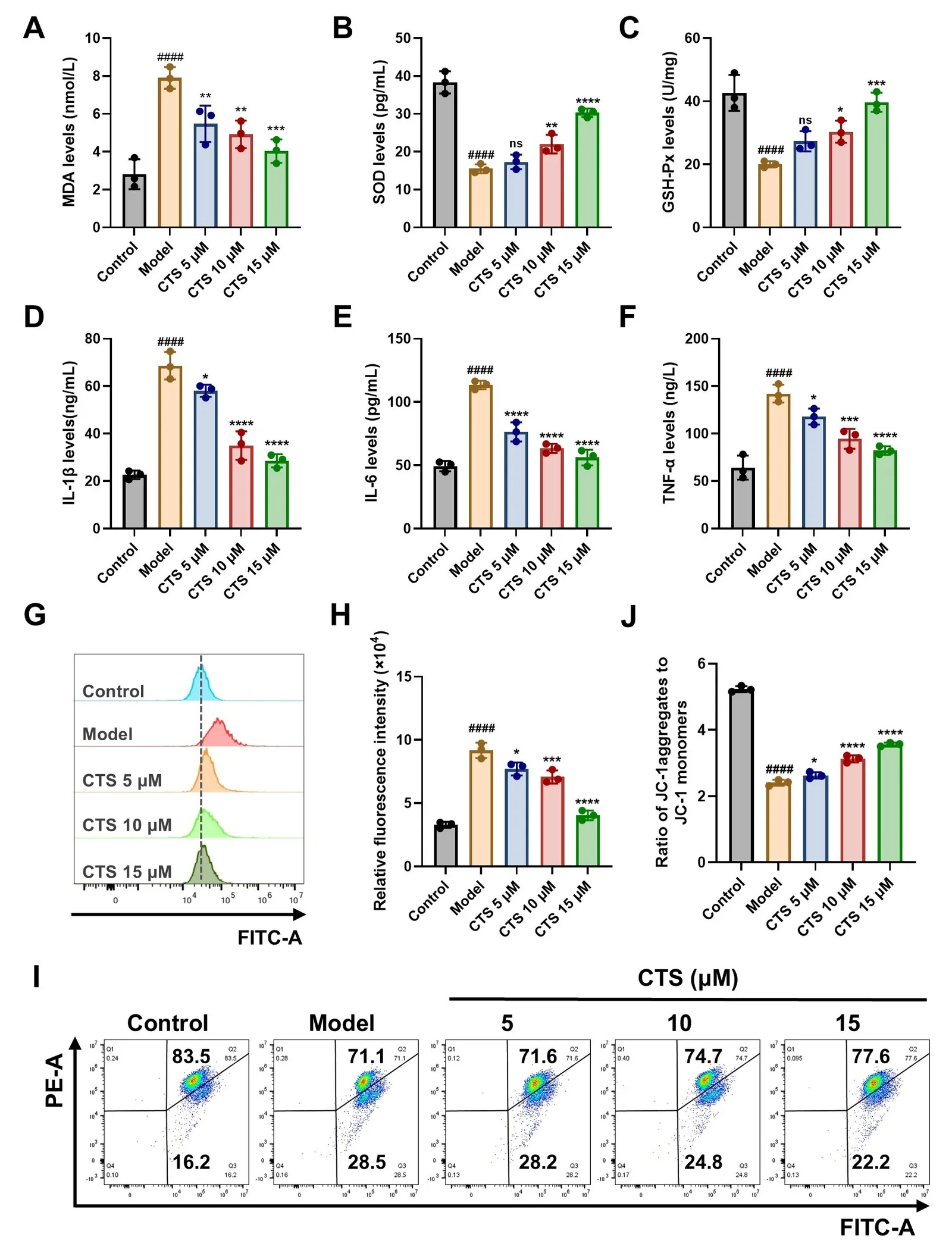
Cryptotanshinone suppresses inflammation and maintains mitochondrial membrane potential stability. MDA (**A**), SOD (**B**), and GSH-Px (**C**) levels in H9C2 cells (*n* = 3). The concentrations of IL-1β (**D**), IL-6 (**E**), and TNF-α (**F**) in H9C2 cells detected by ELISA kits (*n* = 3). (**G**) Intracellular ROS levels measured by flow cytometry. (**H**) Quantitative analysis of the ROS fluorescence intensity (*n* = 3). (**I**) Mitochondrial membrane potential of H9C2 cells detected by the JC-1 probe. (**J**) JC-1 aggregate-to-monomer ratio (*n* = 3). Data are presented as mean ± standard deviation. #—significant difference versus control group; *—significant difference versus model group. * *p* < 0.05, ** *p* < 0.01, *** *p* < 0.001, ####/**** *p* < 0.0001, ns indicates no statistical significance.

Ang II, the chief mediator of the renin–angiotensin system, drives ROS generation via NADPH oxidase (NOX) activation, thereby contributing to cardiac damage [26]. Cardiac hypertrophy is tightly linked to oxidative stress-excessive ROS accumulation and impaired antioxidant defenses trigger cytotoxic effects in cardiomyocytes [27]. Superoxide dismutase (SOD) activity reflects cellular antioxidant capacity, and malondialdehyde (MDA) indicates lipid peroxidation severity. Decreased SOD and elevated MDA collectively signify oxidative–antioxidant imbalance [28,29]. We therefore employed ELISA to evaluate CTS effects on these markers. Compared with control groups, Ang II-treated cardiomyocytes exhibited a 1.81-fold increase in MDA levels, while SOD activity declined by 59.54% and glutathione peroxidase (GSH-Px) activity dropped by 53.13% (Figure 4A–C). CTS dose-dependently reversed these changes, with the highest dose reducing MDA by 48.97%, restoring SOD activity to 79.08% of control levels, and elevating GSH-Px by 98.33% relative to the Ang II group (Figure 4A–C). Inflammatory profiling revealed that Ang II significantly elevated IL-1β, IL-6 and TNF-α, whereas CTS effectively suppressed this pro-inflammatory surge (Figure 4D–F), suggesting that CTS mitigates hypertrophy-associated inflammation. Intracellular ROS, quantified by flow cytometry, were markedly elevated by Ang II treatment, with mean fluorescence intensity increasing by 1.79-fold relative to controls. CTS suppressed this ROS burst in a dose-dependent fashion, achieving a maximal reduction of 55.96% (Figure 4G,H). Mitochondrial membrane potential was then monitored using JC-1 staining (Figure 4I). Ang II increased green monomeric fluorescence and decreased red aggregated fluorescence, yielding a red-to-green ratio decline of 53.98%, indicative of mitochondrial depolarization. CTS restored mitochondrial membrane potential by reversing these shifts, improving the red-to-green ratio by 47.92% over the Ang II group (Figure 4J). Collectively, these quantitative data demonstrate that CTS enhances mitochondrial function, curbs oxidative stress, and restrains inflammatory responses in Ang II-stimulated H9C2 cardiomyocytes.

### 3.4. SIRT3 Knockout Exacerbates Cardiac Hypertrophy

**Figure 5:**
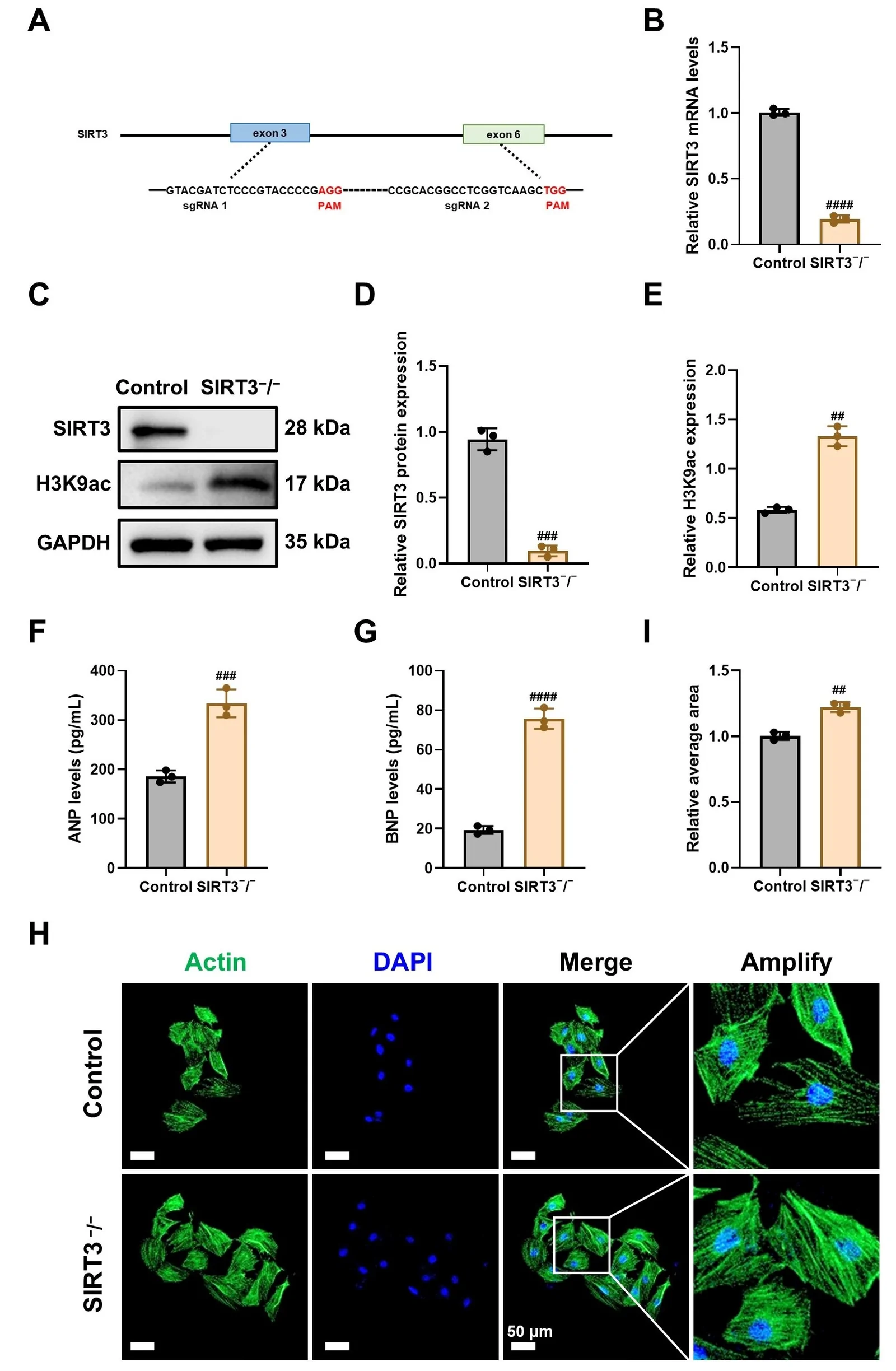
SIRT3 knockout exacerbates cardiac hypertrophy. (**A**) Schematic illustration of the CRISPR/Cas9-mediated SIRT3 knockout. (**B**) SIRT3 mRNA levels detected by real-time qPCR (*n* = 3). (**C**) SIRT3 and H3K9ac protein levels analyzed by Western blot assay. Quantitative analysis of SIRT3 (**D**) and H3K9ac (**E**) levels using ImageJ software (*n* = 3). ANP (**F**) and BNP (**G**) levels in H9C2 cells measured by ELISA (*n* = 3). (**H**) H9C2 cardiomyocyte surface detected by Phalloidin-iFluor 488 Actin-Tracker fluorescent probes (green: actin, blue: nucleus). (**I**) Quantitative analysis of H9C2 cardiomyocyte surface area (*n* = 3). Data are presented as mean ± standard deviation. #—significant difference versus control group. ## *p* < 0.01, ### *p* < 0.001, #### *p* < 0.0001.

As a protein deacetylase, Sirtuin 3 (SIRT3) plays an important role in tolerance to oxidative stress [30]. In addition, SIRT3 has been found to inhibit the acetylation of histone H3K9 (H3K9ac) [31]. To dissect the contribution of SIRT3-mediated histone deacetylation in cardiac hypertrophy, we employed CRISPR-Cas9 to generate a SIRT3-knockout H9C2 cell line (Figure 5A). RT-qPCR assay showed that SIRT3 mRNA level was significantly reduced in the knockout group compared with wild-type controls, verifying efficient knockout (Figure 5B). The successful knockout was also confirmed by Western blot results (Figure 5C,D). Furthermore, SIRT3 knockout was found to elevate histone H3K9 acetylation by 1.28-fold relative to controls (Figure 5E), confirming SIRT3′s role as the dominant deacetylase for this histone mark. ELISA further demonstrated that SIRT3 deletion increased ANP protein levels by 79.54% (Figure 5F) and BNP protein levels by 2.91-fold (Figure 5G) compared with wild-type cells. Actin-Tracker probe detection showed that SIRT3-deficient H9C2 cells exhibited a significantly larger surface area (Figure 5H), with mean cell size increased by 21.92% versus wild-type controls (Figure 5I). Collectively, these data indicate that SIRT3 deficiency increases cell surface area and upregulates hypertrophic markers, underscoring its negative regulatory role in the pathogenesis of myocardial hypertrophy. SIRT3-mediated H3K9ac deacetylation is a potential mechanistic node through which CTS may exert its therapeutic effect.

### 3.5. Cryptotanshinone Regulates Histone Acetylation by Activating SIRT3, Thereby Modulating Mitochondrial Dynamics

**Figure 6:**
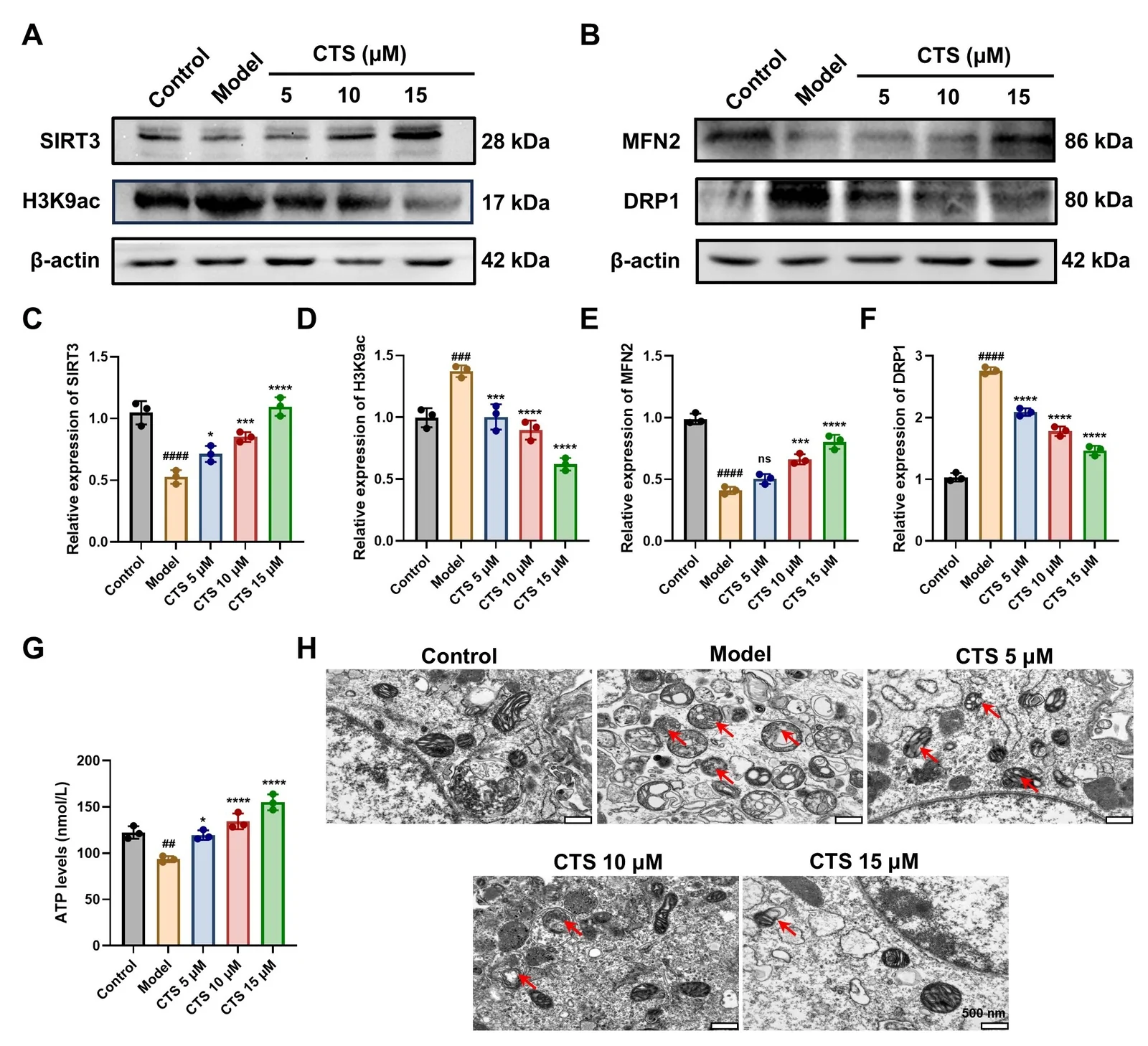
Cryptotanshinone regulates histone acetylation by activating SIRT3, thereby modulating mitochondrial dynamics. (**A**) Effects of CTS on SIRT3 and H3K9ac protein expression detected by Western blot assay. (**B**) MFN2 and DRP1 protein levels in H9C2 cells measured by Western blot assay. Quantitative analysis of SIRT3 (**C**), H3K9ac (**D**), MFN2 (**E**), and DRP1 (**F**) levels (*n* = 3). (**G**) ATP content of H9C2 cells after CTS treatment (*n* = 3). (**H**) Mitochondrial morphology revealed by transmission electron microscopy; red arrows indicate mitochondrial structural abnormalities. Data are presented as mean ± standard deviation. #—significant difference versus control group; *—significant difference versus model group. * *p* < 0.05, ## *p* < 0.01, ###/*** *p* < 0.001, ####/**** *p* < 0.0001, ns indicates no statistical significance.

Building on the previous findings, we hypothesized that CTS regulates mitochondrial homeostasis by upregulating SIRT3 and deacetylating histone H3K9. Mitochondria, the cellular energy factories, are central to cardiomyocyte metabolism and function [8]. To determine whether CTS restores mitochondrial dynamics via the SIRT3/H3K9ac axis, Western blot assay was employed to analyze the effects of CTS on relevant proteins in angiotensin II-treated H9C2 cells (Figure 6A,B). Ang II reduced SIRT3 protein expression by 49.68% relative to controls (Figure 6C), concomitant with a 37.79% elevation in H3K9ac acetylation (Figure 6D), confirming the inverse relationship between SIRT3 activity and this histone mark. Ang II also decreased the fusion protein MFN2 (Figure 6E) and increased the fission protein DRP1 (Figure 6F), shifting the balance toward excessive fission and compromising energy metabolism. However, CTS reversed these alterations in a concentration-dependent manner: at the highest dose, SIRT3 expression was increased by 1.08-fold (Figure 6C), H3K9ac was reduced by 54.85% (Figure 6D), MFN2 was upregulated by 95.93% (Figure 6E), and DRP1 was suppressed by 46.92% (Figure 6F). Similarly, ELISA results showed that, as the dose of CTS increased, MFN2 was upregulated and DRP1 was downregulated in the cardiac hypertrophy model (Figure S6). Disruption of mitochondrial dynamics affects cellular ATP production, and it was further found that Ang II lowered the ATP content but CTS dose-dependently restored ATP levels in H9C2 cells (Figure 6G). Moreover, transmission electron microscopy revealed that the control group’s mitochondria displayed intact double-layered membranes with closely packed cristae, whereas Ang II-treated cells exhibited swollen organelles with fragmented, sparse and indistinct cristae. CTS intervention improved mitochondrial morphology, restoring cristae integrity and reducing membrane damage (Figure 6H). Taken together, these findings demonstrate that CTS upregulates SIRT3 and reduces H3K9ac levels. These epigenetic changes are correlated with a rebalancing of DRP1/MFN2-mediated mitochondrial dynamics, resulting in enhanced energy metabolism and structural integrity in Ang II-stimulated cardiac hypertrophy.

### 3.6. SIRT3 Inhibition Eliminates the Therapeutic Effect of Cryptotanshinone

**Figure 7:**
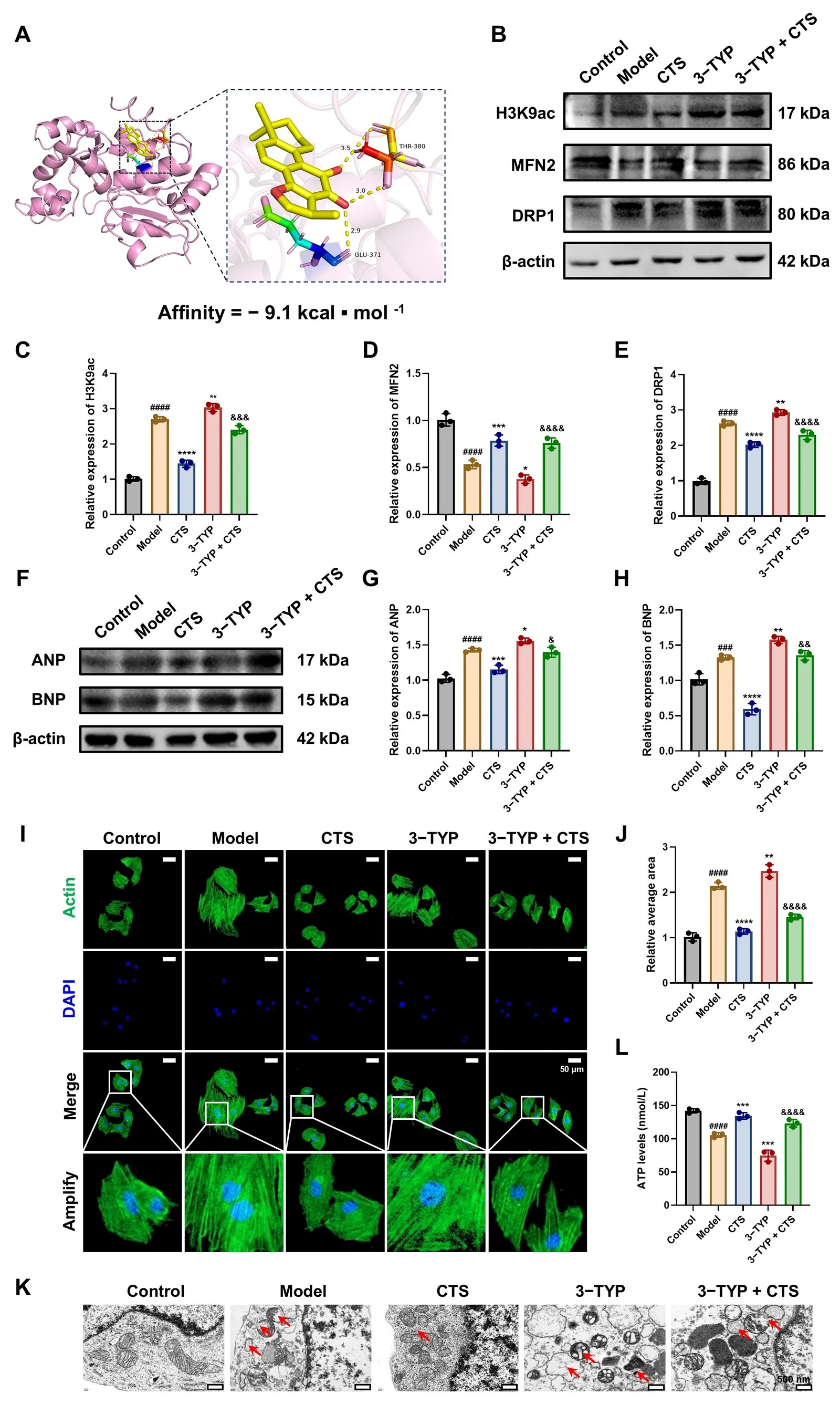
SIRT3 inhibition eliminates the therapeutic effect of cryptotanshinone. (**A**) Molecular docking simulation of CTS with SIRT3. (**B**) Effects of different treatments on H3K9ac, MFN2, and DRP1 expression detected by Western blot assay. Quantitative analysis of H3K9ac (**C**), MFN2 (**D**), and DRP1 (**E**) levels using ImageJ software (*n* = 3). (**F**) Western blot images for the hypertrophic markers ANP and BNP. Quantitative analysis of ANP (**G**) and BNP (**H**) contents (*n* = 3). (**I**) Cell surface area measured by Phalloidin-iFluor 488 Actin-Tracker probe (green: actin, blue: nucleus). (**J**) Quantitative analysis of H9C2 cardiomyocyte surface area (*n* = 3). (**K**) TEM images of mitochondrial morphology; red arrows indicate mitochondrial structural abnormalities. (**L**) ATP levels of H9C2 cells under different treatment (*n* = 3). Data are presented as mean ± standard deviation. #—significant difference versus control group; *—significant difference versus model group; &—significant difference versus 3-TYP group. */& *p* < 0.05, **/&& *p* < 0.01, ###/***/&&& *p* < 0.001, ####/****/&&&& *p* < 0.0001.

Our previous findings demonstrated that CTS alleviates cardiac hypertrophy through SIRT3-mediated histone deacetylation. Molecular docking simulation predicted a strong interaction between CTS and SIRT3, with a binding energy of −9.1 kcal mol^−1^ (Figure 7A). To further validate this mechanism at the functional level, the SIRT3 inhibitor 3-TYP was employed. Following Ang II-induced cardiac hypertrophy, 3-TYP was co-administered with CTS. Western blot analysis revealed that compared with the Ang II model group, 3-TYP administration further exacerbated histone H3K9 acetylation. CTS co-treatment reduced H3K9ac levels by 20.75% relative to the 3-TYP group, indicating that CTS counteracts Ang II-induced hypertrophy by enhancing SIRT3 deacetylase activity (Figure 7B,C). Mitochondrial dynamics mirrored these changes: 3-TYP disrupted the DRP1/MFN2 balance, driving excessive mitochondrial fission. In contrast, CTS restored fusion–fission equilibrium, reflected by a 2.02-fold upregulation of MFN2 and a 21.62% downregulation of DRP1 in the 3-TYP + CTS group relative to the 3-TYP group (Figure 7D,E). Cardiac hypertrophy protein markers exhibited the same pattern: ANP and BNP were elevated in the 3-TYP group, while CTS co-administration reduced ANP by 10.28% and BNP by 13.95% (Figure 7F,G), confirming that SIRT3 inhibition exacerbates hypertrophic injury, whereas CTS rescues this phenotype. H9C2 cell surface area corroborated this trend: 3-TYP treatment expanded mean cell surface area by an additional 15.24% over the Ang II group, while CTS co-treatment reduced it by 41.03% (Figure 7I,J). Transmission electron microscopy revealed that control cardiomyocyte mitochondria displayed compact structures with orderly arrangement and intact cristae. Ang II induced mitochondrial membrane rupture and cristae loss, 3-TYP exacerbated these defects, and combined CTS treatment restored mitochondrial cristae density (Figure 7K). What is more, ATP content was lowest in the 3-TYP group, declining to 52.59% of control levels, consistent with aggravated mitochondrial dysfunction. CTS restored ATP levels to 86.84% of control values, consistent with its capacity to scavenge reactive oxygen species, alleviate oxidative stress, and enhance mitochondrial function (Figure 7L). Collectively, the 3-TYP inhibitor studies further validate that CTS exerts cardioprotective effects by activating SIRT3 deacetylase activity, preserving mitochondrial integrity, and mitigating hypertrophic and oxidative damage.

## 4. Discussion

This study provides evidence that CTS exerts potent anti-hypertrophic effects through SIRT3 activation. These effects are accompanied by reduced H3K9ac levels and the restoration of DRP1/MFN2 balance. Our integrated investigation delineates a novel mechanistic framework wherein CTS restores mitochondrial dynamic equilibrium and attenuates oxidative stress, inflammation, and apoptosis. These findings position CTS as a promising therapeutic candidate for cardiac hypertrophy.

Our results demonstrate that SIRT3 is a critical molecular target of CTS. The inhibition of SIRT3 not only intensified the pathological features of Ang II-induced hypertrophy but also exacerbated mitochondrial dysfunction, characterized by excessive fission and cristae disorganization. This observation aligns with work establishing SIRT3 as an essential mitochondrial fidelity protein that maintains organelle function and cardiac homeostasis [32]. A particularly novel observation in this study is the coordinated changes in SIRT3 activity and H3K9ac levels concurrent with the regulation of mitochondrial dynamics. We demonstrate that SIRT3 deficiency leads to H3K9 hyperacetylation, suggesting a potential mechanistic link between a mitochondrial deacetylase and the nuclear transcriptional control of mitochondrial fission/fusion genes. Histone modifications have been found to regulate the expression of DRP1 and MFN2 [33]. The subsequent restoration of the DRP1/MFN2 balance by CTS, shifting mitochondrial dynamics away from pathological fission and towards homeostatic fusion, represents a downstream convergence point for its therapeutic actions. Mitochondrial fragmentation, driven by DRP1 upregulation and MFN2 downregulation, is a hallmark of cardiac hypertrophy and heart failure [34]. CTS corrected this imbalance, with concomitant recovery of mitochondrial ultrastructure, membrane potential, and ATP synthesis. These findings are in excellent agreement with recent studies highlighting the therapeutic potential of targeting mitochondrial dynamics in cardiovascular diseases [35]. Furthermore, our data show that the anti-apoptotic effects of CTS, manifested by the normalization of the Bax/Bcl-2 ratio and caspase-3 cleavage, are downstream consequences of this mitochondrial protection, corroborating the central role of mitochondrial integrity in determining cell fate [36]. The translational potential of CTS is underscored by its pleiotropic effects, which simultaneously target the interconnected pathologies of oxidative stress and inflammation. ROS and cytokine levels are important markers that not only affect cell viability but also influence the immune microenvironment, thereby playing a role in disease progression [37]. We observed that CTS reduced Ang II-induced ROS and pro-inflammatory cytokines. This multifaceted cardioprotection is a significant advantage over strategies targeting a single downstream node, as the complex pathophysiology of cardiac hypertrophy involves a series of oxidative stress and inflammation.

The findings of this study establish a compelling foundation for further translational and mechanistic exploration. Building on the robust anti-hypertrophic effects of CTS demonstrated in the Ang II infusion mouse model and H9c2 cardiomyocytes, future investigations will extend these observations to chronic pressure-overload models, thereby evaluating the therapeutic efficacy of CTS in settings that more closely recapitulate clinical heart failure. Furthermore, having observed a correlation between SIRT3-mediated H3K9 deacetylation and the expression of DRP1 and MFN2, subsequent studies will focus on delineating the specific transcription factors and co-regulatory complexes recruited to these gene promoters, which will provide deeper insight into the mitochondrial–nuclear crosstalk that drives pathological cardiac remodeling. In addition, given the functional interplay among the sirtuin family, future work will explore the potential coordinated or compensatory roles of other isoforms, particularly SIRT1, in the therapeutic mechanism of CTS. These multifaceted investigations will not only refine our understanding of CTS pharmacology but also accelerate the development of SIRT3-targeted therapeutic strategies for cardiac hypertrophy.

## 5. Conclusions

In summary, CTS exhibits potent therapeutic efficacy against cardiac hypertrophy. Our findings demonstrate that CTS mitigates pathological remodeling through multimodal mechanisms: (i) restoring mitochondrial energy metabolism and structural integrity by upregulating SIRT3, which is associated with decreased H3K9ac and a rebalancing of DRP1/MFN2-mediated fission–fusion dynamics, and (ii) attenuating oxidative stress, inflammation, and cardiomyocyte apoptosis triggered by chronic Ang II exposure. These results shed light on the cardioprotective mechanisms of CTS, establishing it as a promising therapeutic candidate for preventing hypertrophy-associated heart failure.

## Data Availability

All data supporting the findings of this study are included in this published article and its Supplementary Materials. Further inquiries can be directed to the corresponding author(s).

## Supplementary Materials

The following supporting information can be downloaded at https://www.mdpi.com/article/10.3390/antiox15101329/s1. Figure S1: Heart-weight-to-body-weight ratio (HW/BW); Figure S2: H&E staining of liver, spleen, lung, and kidney from all groups; Figure S3: Changes in inflammatory factors in myocardial tissue from each group; Figure S4: ANP and BNP levels in H9C2 cells measured by ELISA; Figure S5: Hoechst staining of H9C2 cell nucleus; Figure S6: MFN2 and DRP1 levels in cardiac tissue homogenate measured by ELISA; Table S1: Primer sequences for quantitative real-time PCR; Table S2: sgRNA sequences for gene knockout.

## Abbreviations

The following abbreviations are used in this manuscript:

Ang II	Angiotensin II
ANP	Atrial natriuretic peptide
ATP	Adenosine triphosphate
BNP	Brain natriuretic peptide
CTS	Cryptotanshinone
DRP1	Dynamin-related protein 1
ELISA	Enzyme-linked immunosorbent assay
H3K9ac	Histone H3 acetylated at lysine 9
IL	Interleukin
MDA	Malondialdehyde
MFN2	Mitofusin 2
MMP	Mitochondrial membrane potential
ROS	Reactive oxygen species
SIRT3	Sirtuin 3
SOD	Superoxide dismutase
TEM	Transmission electron microscope
TNF-α	Tumor necrosis factor-alpha
