# Supplementary material for: Cryptotanshinone Alleviates Cardiac Hypertrophy Through SIRT3-Mediated Epigenetic Regulation and Mitochondrial Dynamics
Source: Antioxidants (Basel). 2026 Oct 15;15(10):1329. doi: 10.3390/antiox15101329 (PMC13701624; doi:10.3390/antiox15101329)
Supplement: Supplementary file 1 [file antioxidants-15-01329-s001.zip › antioxidants-4593513-supplementary.pdf]

Cryptotanshinone alleviates cardiac hypertrophy through SIRT3−mediated epigenetic regulation and mitochondrial dynamics

Jianming Xing [1,2,3,4,†], Peng Ji [2,3,†], Xintong Gu [2,3], Shuoxi Guo [2,3], Meilun Wang [2,3], Xushuang Jia [3,4], Jiaqi Chen [3,4], Junjie Chang [3], Xiaohan Cui [3], Ye Jin [4], Haicheng Gao [1*], Da Liu [2,3*]

1 Department of Clinical Pharmacy, School of Pharmaceutical Sciences, Jilin University, Changchun 130021, China

2 Public Laboratory Center, Changchun University of Chinese Medicine, Changchun 130117, China

3 Department of Pharmacy, Changchun University of Chinese Medicine, Changchun 130117, China

4 Northeast Asian Institute of Traditional Chinese Medicine, Changchun University of Chinese Medicine, Changchun 130117, China

* Correspondence: gaohc@jlu.edu.cn (H.G.); Da Liu: liuda@ccucm.edu.cn (D.L.)

† These authors contributed equally to this work.

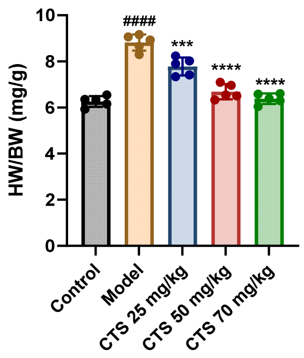

Figure S1. Heart weight to body weight ratio (HW/BW, n=5). Data are presented as mean ± standard deviation. #− significant difference versus control group, *− significant difference versus model group. ###/***P < 0.001, ####/****P < 0.0001.

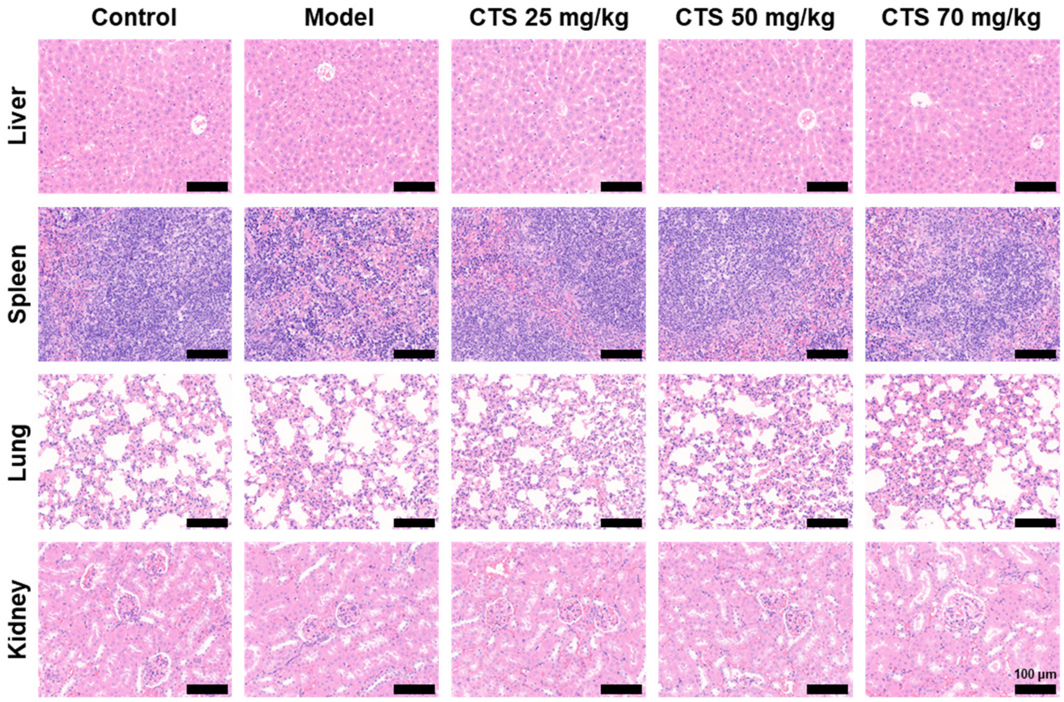

Figure S2. H&E staining of liver, spleen, lung, and kidney from all groups.

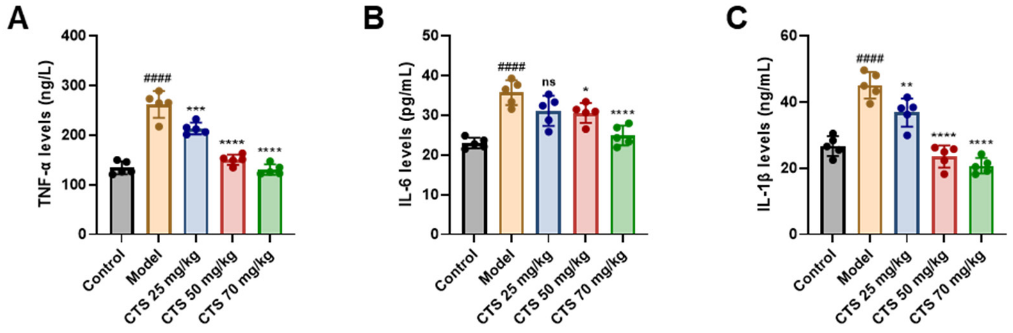

Figure S3. Changes in inflammatory factors in myocardial tissue from each group (n=5). (A) TNF−α; (B) IL−6; (C) IL−1β. Data are presented as mean ± standard deviation. #− significant difference versus control group, *− significant difference versus model group. *P < 0.05, **P < 0.01, ***P < 0.001, ####/****P < 0.0001, ns indicates no statistical significance.

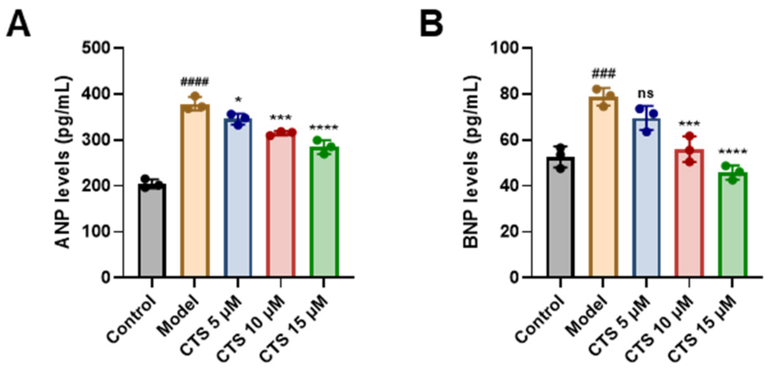

Figure S4. ANP and BNP levels in H9C2 cells measured by ELISA (n=3). (A) ANP; (B) BNP. Data are presented as mean ± standard deviation. #− significant difference versus control group, *− significant difference versus model group. *P < 0.05, ###/***P < 0.001, ####/****P < 0.0001, ns indicates no statistical significance.

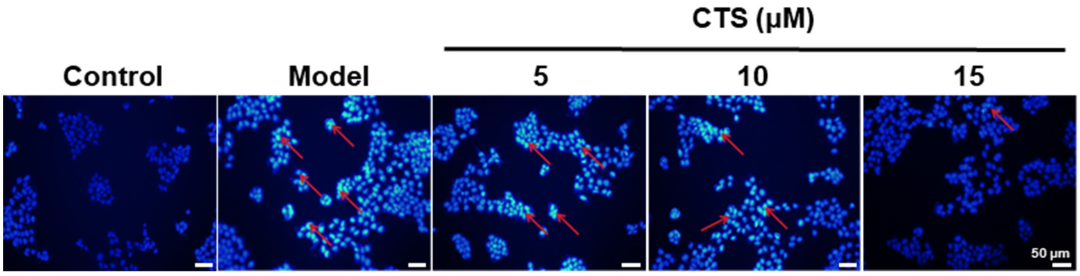

Figure S5. Hoechst staining of H9C2 cell nucleus. Red arrows indicate apoptotic cells.

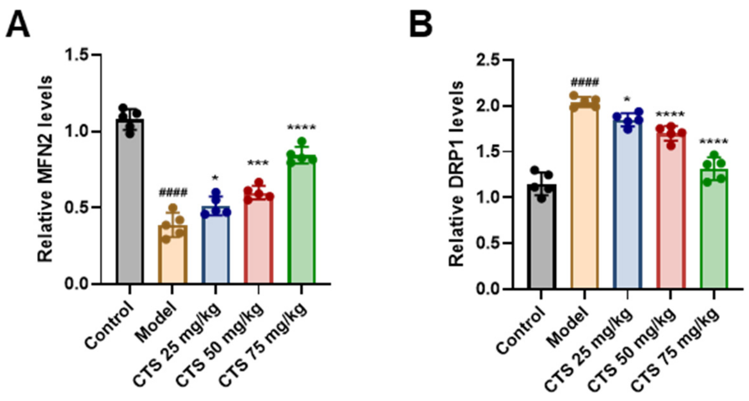

Figure S6. MFN2 and DRP1 levels in cardiac tissue homogenate measured by ELISA (n=5). (A) MFN2; (B) DRP1. Data are presented as mean ± standard deviation. #− significant difference versus control group, *− significant difference versus model group. *P < 0.05, ***P < 0.001, ####/****P < 0.0001.

Table S1. Primer sequences for quantitative real−time PCR.

Primer name	Sequence
SIRT3 forward	5’− ACCCAGTGGCATTCCAGAC −3’
SIRT3 reverse	5’− GGCTTGGGGTTGTGAAAGAAG −3’
GAPDH forward	5’− TGGTATCGTGGAAGGACTCA −3’
GAPDH reverse	5’− GGGCCATCGACAGTCTTC −3’

Table S2. sgRNA sequences for gene knockout.

sgRNA name	Sequence
sgRNA−1 forward	5’− CACCGTACGATCTCCCGTACCCCG −3’
sgRNA−1 reverse	5’− AAACCGGGGTACGGGAGATCGTAC −3’
sgRNA−2 forward	5’− CACCGCCGCACGGCCCTCGGTCAAGC −3’
sgRNA−2 reverse	5’− AAACGCTTGACCGAGGCCCGTGCGG −3’
